# Biodegradation of Single-Walled Carbon Nanotubes in Macrophages through Respiratory Burst Modulation

**DOI:** 10.3390/ijms17030409

**Published:** 2016-03-22

**Authors:** Jie Hou, Bin Wan, Yu Yang, Xiao-Min Ren, Liang-Hong Guo, Jing-Fu Liu

**Affiliations:** 1 State Key Laboratory of Environmental Chemistry and Ecotoxicology, Research Center for Eco-Environmental Sciences, Chinese Academy of Sciences, Beijing 100085, China; jiehou_st@rcees.ac.cn (J.H.); yuyang@rcees.ac.cn (Y.Y.); xmren@rcees.ac.cn (X.-M.R.); jfliu@rcees.ac.cn (J.-F.L.); 2 Institute of Environment and Health, Jianghan University, Wuhan 430056, China

**Keywords:** single-walled carbon nanotubes, biodegradation, respiratory burst, *in vitro* enzymatic system

## Abstract

The biodegradation of carbon nanotubes (CNTs) may be one of major determinants of the toxic outcomes in exposed individuals. In this study, we employed a macrophage/monocyte model, Raw264.7, to investigate the feasibility of regulating the biodegradation of three types of single-walled carbon nanotubes (SWCNTs) (pristine, ox-, and OH-SWCNTs) by respiratory burst modulation. An artificial fluid mimicking the enzymatic reactions of respiratory burst was constituted to reveal the role of respiratory burst played in SWCNT biodegradation. The biodegradation of SWCNTs were characterized by Raman, ultraviolet-visible-near-infrared spectroscopy, and transmission electron microscopy. Our results showed significantly accelerated biodegradation of ox-SWCNTs and OH-SWCNTs in macrophages activated by phorbol myristate acetate (PMA), which could be prevented by *N*-acetyl-l-cysteine (NAC), whereas p-SWCNTs were resistant to biodegradation. Similar tendencies were observed by using the *in vitro* enzymatic system, and the degradation rates of these SWCNTs are in the order of OH-SWCNTs > ox-SWCNTs >> p-SWCNTs, suggesting a pivotal role of respiratory burst in accelerating the biodegradation of SWCNTs and that defect sites on SWCNTs might be a prerequisite for the biodegradation to occur. Our findings might provide invaluable clues on the development of intervention measurements for relieving the side effects of SWCNTs and would help to design safer SWCNT products with higher biodegradability and less toxicity.

## 1. Introduction

Carbon nanotubes (CNTs) are one of the most important and widely used nanomaterials in research fields and consumer industrials [1,2]. Among them, functionalized single-walled carbon nanotubes (f-SWCNTs) are particularly attracting in biomedical applications [3,4], such as biomedical imaging [5], nanoinjectors [6], nanoneuroengineering [7], gene therapy [8] and drug delivery [4,9,10]. Human exposure to CNTs can potentially occur throughout the CNTs lifecycle, ranging from laboratory research, manufacturing, incorporation of CNTs into products, manipulation of CNT containing products, and finally through disposal and recycling of CNTs [11,12,13]. With increasing production and applications of SWCNTs, one can envision an increase of the chance of human exposure to SWCNTs and great concerns are raised on the side effects of CNTs.

Imaging and spectroscopy information unequivocally support the fact that SWCNTs can be taken up, retained, excreted or degraded within nucleated cells [14,15,16]. Study showed that pristine SWCNTs exposed to mice remained in the lungs for over three months [17]. Once inside organisms, the physical and chemical properties of nanomaterials usually undergo a series of alterations that either increase or reduce their toxicity. The internalized CNTs are cytotoxic [18,19,20,21], and subsequently cause pulmonary inflammation, granuloma formation and fibrosis [17,22,23,24] *in vivo*, mostly by inducing oxidative stress [25,26]. Reactive oxygen species (ROS)-mediated cytotoxicity can be induced by the metal impurities present in CNTs, which can be removed through CNT oxidation [27,28]. Carbon nanotubes were once considered to be resistant to chemical damage due to their rigid and perfect chemical structure, which rendered them immune to biodegradation. However, functionalized SWCNTs carrying defect sites on the sidewall and tips of the carbon nanotubes have been demonstrated to be degraded by plant-derived horse radish peroxidase (HRP) [29,30]. Thorough disintegration of the carbon nanotubes was achieved in 10 days, and oxidized aromatic fragments were detected in the course of nanotube degradation, which eventually evolved to carbon dioxide [31,32]. A study investigated the degradation of SWCNTs and MWCNTs in artificial fluids mimicking phagolysosome content and found that carboxylated SWCNTs were degraded over time along with the fracture of CNTs and disaggregation of bundles, further proving the feasibility of biodegradation of CNTs in mammalian cells [33,34]. Indeed, neutrophil myeloperoxidase (MPO) and eosinophil peroxidase (EPO) were both demonstrated to be able to degrade CNTs after supplement of exogenous H_2_O_2_ [35,36]. Reactive intermediates of the peroxidase-mediated reactions were found to play an important role in the process of oxidative biodegradation of nanotubes [35,36,37]. Also nanotubes can be degraded by the lignin peroxidase [38] or bacteria [39], even the neutrophil extracellular traps (NETs) [40]. Most importantly, biodegraded SWCNTs did not generate an inflammatory response when aspirated into the lungs of mice [35]. Therefore, the capability of regulating the biodegradation of CNTs is of significance in modulating their toxicity.

In mammalian immune systems, polymorphonuclear neutrophils (PMN) have relatively higher level of MPO than macrophages do, and thus are more efficient in degrading CNTs [35,36]. However, it is macrophages instead of PMN cells that are more practically relevant in digesting CNTs due to: (1) PMN recruitment after SWCNT exposure is a transient rather than persistent inflammatory response, and there is no strong evidence for SWCNT phagocytosis by PMN; (2) macrophages are distributed almost throughout the body, scrutinizing for foreign particles, including nanomaterials, so that they have better chance of encountering and engulfing CNTs; (3) there are multiple evidences showing that CNTs resident in tissues including lung and brain were engulfed by macrophages [41,42,43]. Fortunately, monocytes/macrophages can be activated as they come into contact with bacteria or fungi, and the oxygen consumption increases significantly along with the rapid generation of reactive oxygen species (superoxide radicals and hydrogen peroxide) [35,38] to degrade internalized particles and bacteria, which is defined as respiratory burst [44]. The whole reaction is initiated with the activation of nicotinamide adenine dinucleotide phosphate (NADPH)-oxidase that reduces O_2_ to superoxide anion (O_2_*^−^), which is then catalyzed by superoxide dismutase (SOD) to form H_2_O_2_. Myeloperoxidase (MPO) further combines H_2_O_2_ with Cl^−^ to produce hypochlorite, which plays a role in destroying bacteria as well as internalized particles such as SWCNTs [35,44,45]. Chemicals such as lipopolysaccharide (LPS), PMA, are known to initiate the respiratory burst in macrophages, and thus provide a meaningful way to modulate the biodegradation of CNTs.

In this study, we employed a macrophage/monocyte model, Raw264.7 cells, to investigate the feasibility of controlling the biodegradation of SWCNTs, by either respiratory burst inducer or the anti-oxidant treatment. In addition, an artificial fluid was constructed to simulate the enzymatic reactions of respiratory burst to reveal the role of respiratory burst played in the SWCNT biodegradation. Simultaneously, different SWCNTs (pristine, acid oxidized and hydroxylated SWCNTs) were used to study the influence of functional surface groups on the biodegradation of SWCNTs. The results showed that stimulation of respiratory bursts in macrophages could accelerate the degradation of SWCNTs significantly, while anti-oxidant treatment inhibited the process. Pristine SWCNTs were resistant to degradation, whereas acid oxidized and hydroxylated SWCNTs were degraded upon respiratory bursts, indicating that functionalization of SWCNTs introducing defect sites is a prerequisite for CNT degradation.

## 2. Results

### 2.1. Characterization of Single-Walled Carbon Nanotubes (SWCNTs)

**Figure 1:**
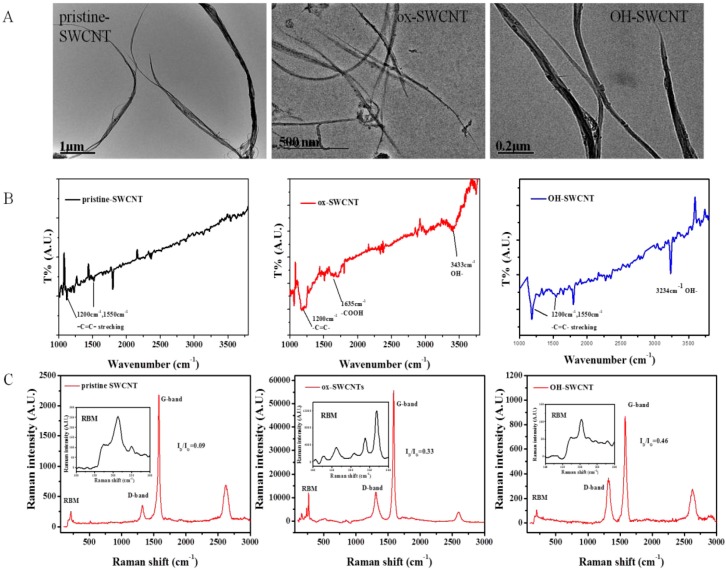
Characterization of three types of SWCNTs: (**A**) TEM images of (from left to right) pristine-SWCNTs, ox-SWCNTs, and OH-SWCNTs; (**B**) FT-IR spectra of (from left to right) pristine-SWCNTs, ox-SWCNTs, and OH-SWCNTs, showing the presence of –C=C– stretching (υ = 1200 and 1550 cm^−1^), –COOH (υ = 1635 cm^−1^) and –OH (υ = 3200–3600 cm^−1^) [46,47,48]; (**C**) Raman spectra of (from left to right) pristine-SWCNTs, ox-SWCNTs, and OH-SWCNTs. G-band and D-band were indicated. I_D_/I_G_ ratios were shown as insets.

The characterization of SWCNTs by transmission electron microscopy (TEM) showed that pristine SWCNTs (p-SWCNTs), acid-oxidized SWCNTs (ox-SWCNTs), and hydroxylated SWCNTs (OH-SWCNTs) all presented as fibrillar shape and retained the structural integrity of carbon nanotubes (Figure 1A). Fourier transform infrared spectroscopy (FT-IR) analyses on these SWCNTs showed the presence of carboxyl groups (υ = 1635 cm^−1^, –COOH) and hydroxyl groups (υ = 3433 cm^−1^, –OH) on the surface of ox-SWCNTs, while only hydroxyl groups (υ = 3234 cm^−1^, –OH) were present on the surface of OH-SWCNTs and no functional group was found on the surface of p-SWCNTs (Figure 1B). In the Raman spectrum, the two characteristic peaks: disorder induced D-band at ~1340 cm^−1^ (±10) and tangential mode G-band at ~1580 cm^−1^ (±10) were identified and I_D_/I_G_ ratios were shown as insets (Figure 1C) in the order of: OH-SWCNTs (I_D_/I_G_ = 0.46) > ox-SWCNTs (0.33) > p-SWCNTs (0.09). The diameters and length of these SWCNTs were listed in supplementary Table S1.

### 2.2. Biodegradation of SWCNTs in Macrophages

#### 2.2.1. Induction of Respiratory Burst in Macrophages

**Figure 2:**
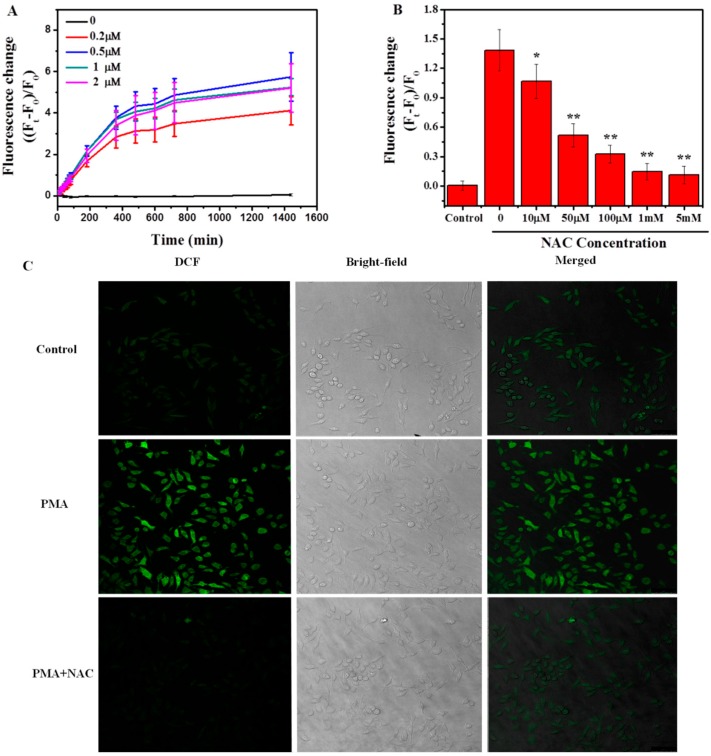
Induction and inhibition of respiratory burst in macrophages by PMA and NAC, respectively, evaluated through the generation of ROS. (**A**) Fluorescence intensity change of DCF over time in macrophages under the treatment of 0–2 μM PMA; (**B**) Fluorescence intensity change of DCF in macrophages with different concentrations of NAC pretreatment, followed by 0.5 μM PMA stimulation for 3 h (Ft); (**C**) Representative confocal images of macrophages treated in (**B**) with PMA concentration 0.5 μM, and NAC 1 mM. Green fluorescence represents DCF signal. The control group was cells without any treatment. Scale bar = 50 μM. (* *p* < 0.05; ** *p* < 0.01).

The respiratory burst can be activated through the interaction between macrophages and microorganisms or by other stimuli including chemotactic factors and phorbol esters such asphorbol myristate acetate (PMA) [49,50,51]. In this study, we used PMA as the activator to induce respiratory burst in macrophages, and the anti-oxidant, *N*-acetyl-l-cysteine (NAC), as the inhibitor of the respiratory burst. The respiratory burst was evaluated through the measurement of reactive oxygen species (ROS) generation by using a 2′,7′-dichlorofluorescein diacetate (DCF-DA) assay. When cells were treated with different concentrations of PMA (0–2 μM), the fluorescence of intracellular DCF increased over time and 0.5 μM PMA has the highest intensity of DCF fluorescence, indicating the maximum generation of ROS and respiratory burst in macrophages under this treatment (Figure 2A). We further optimized the concentration of NAC pretreatment for inhibiting the respiratory burst. The results showed that NAC pretreatment could significantly inhibit the ROS production in a concentration-dependent manner (Figure 2B). One mM NAC pretreatment was selected for further experiments. In addition, the effects of PMA and NAC on the generation of ROS in macrophages were observed by direct imaging of DCF fluorescence signals in cells and are shown in Figure 2C.

#### 2.2.2. Characterization of SWCNTs Degradation in Macrophages

**Figure 3:**
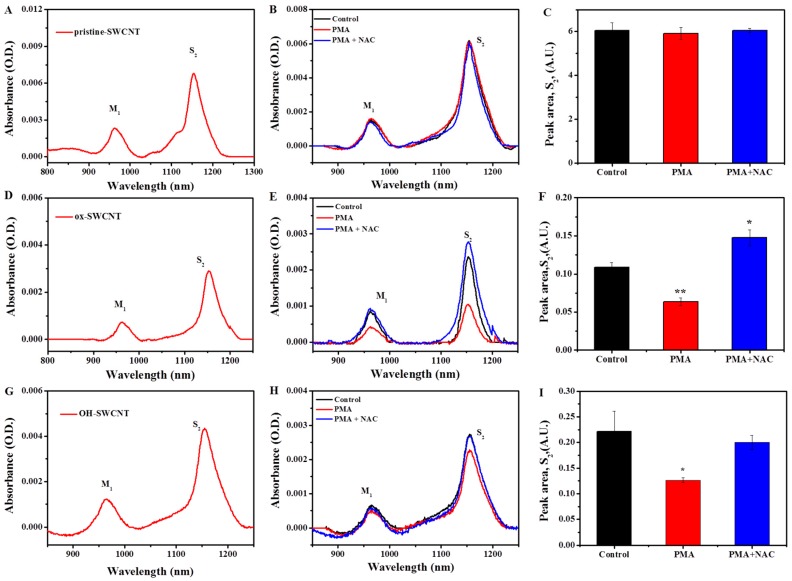
Biodegradation of SWCNTs in macrophages for 24 h with or without PMA activation, as evaluated by the infrared (NIR) spectra, normalized against baseline. (**A**,**D**,**G**) Typical UV-vis-NIR spectra of p-SWCNTs, ox-SWCNTs and OH-SWCNTs, respectively; (**B**,**E**,**H**) Influence of PMA and NAC treatment on the absorbance of M_1_ and S_2_ bands of p-SWCNTs, ox-SWCNTs and OH-SWCNTs in macropahges, respectively. Black line: control macrophages (untreated); Red line: PMA-activated macrophages; Blue line: PMA-activated macrophages with NAC pretreatment; (**C**,**F**,**I**) Quantification of the peak area of characteristic semiconducting transition band (S_2_) of (**C**) p-SWCNTs; (**F**) ox-SWCNTs; and (**I**) OH-SWCNTs in macrophages. Results were from at least three independent experiments and expressed as mean ± S.D. (* *p* < 0.05; ** *p* < 0.01).

On the basis of the respiratory burst model, we exposed the macrophages to different types of SWCNTs and observed the degradation of these SWCNTs by using transmission electron microscopy (TEM), ultraviolet-visible-near-infrared (UV-vis-NIR) spectroscopy, and Raman spectroscopy [31]. The optical absorption of carbon nanotubes is a quick and facile method to obtain the information of nanotubes [52]. Because the SWCNTs are usually synthesized in the format of mixtures with various chiralities and diameters, they possess metallic and semiconducting properties. Since respiratory burst takes place mainly inside of cells, CNTs must be internalized first before degradation occurs, as evidenced in the present study (Figure S1). The treatment of p-SWCNTs, ox-SWCNTs, and OH-SWCNTs (5 µg/mL) in the presence or absence of PMA (0.5 µM) did not cause cell viability loss (Figure S2). In this study, we observed the typical UV-vis-NIR spectra of carbon nanotubes, showing the characteristic metallic band (M_1_, ~900–1000 nm) and semiconducting transition absorbing band (S_2_, ~1100–1200 nm) [53] (Figure 3A,D,G). The transitions related to the sp^2-^ carbon framework of the SWCNTs will lessen when the oxidation of the graphitic structure occurs, and decreased absorbance of the band would indicate the degradation of carbon nanotubes [32]. We found a decrease in the absorbance of the metallic, especially the semiconducting band of SWCNTs in macrophages treated with PMA (Figure 3E,H, red lines), compared to those in untreated macrophages (black lines). After normalization against baseline and through the peak area calculation, we quantitatively detected the significant decrease in S_2_ band of ox- and OH-SWCNTs in PMA-treated macrophages (Figure 3F,I), suggesting different degrees of degradation of both SWCNTs. Interestingly, when cells were pretreated with 1 mM NAC, followed by PMA stimulation, the decreased absorbance of S_2_ band was alleviated significantly for OH-SWCNTs, and the even the absorbance was increased for ox-SWCNTs (Figure 3E,H, blue line), suggesting that anti-oxidant could inhibit the degradation of SWCNTs. No increase or decrease of either M_1_ or S_2_ band absorbance was observed for p-SWCNTs (Figure 3B,C), suggesting that p-SWCNTs are resistant to the biodegradation by cells, even when macrophages were in respiratory burst.

**Figure 4:**
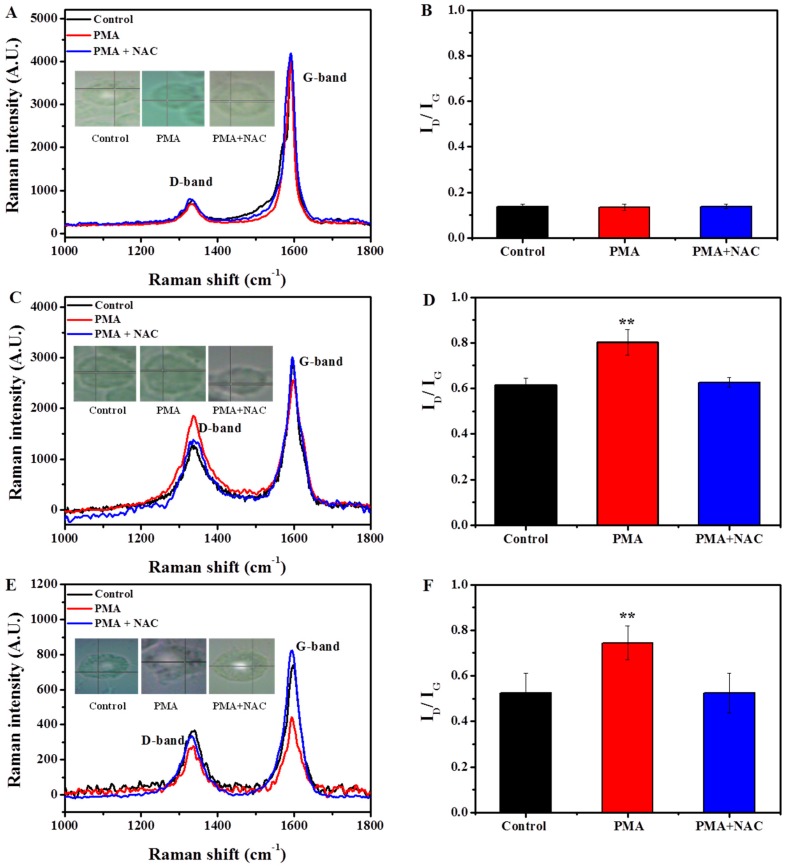
Biodegradation of SWCNTs by macrophages for 24 h, as characterized with Raman spectra. (**A**,**C**,**E**) Representative Raman spectra of p-SWCNTs, ox-SWCNTs, and OH-SWCNTs incubated with un-stimulated macrophages (control, black line), PMA-activated macrophages (PMA, red line), and PMA-activated macrophages with NAC treatment (PMA + NAC, blue line) for 24 h. Corresponding cell images and sampling sites were shown as insets; (**B**,**D**,**F**) Quantitative I_D_/I_G_ ratios of p-SWCNTs, ox-SWCNTs, and OH-SWCNTs treated in (**A**,**C**,**E**), respectively. The results were obtained from at least 20 spectra and expressed as mean ± S.D. (standard deviation). (** *p* < 0.01).

The damage of SWCNT structure was also characterized by Raman microscopy (Figure 4), which allows monitoring the evolution of the oxidation of the SWCNTs [33]. Compared to their corresponding original SWCNTs, ox-SWCNTs and OH-SWCNTs showed increased intensity of disorder induced D-band at ~1340 cm^−1^ (±10) whereas the tangential mode G-band at ~1580 cm^−1^ (±10) was decreased in untreated macrophages, indicating the oxidation damage on the graphene walls and the resulting of I_D_/I_G_ ratios were increased from 0.33 to 0.63 (±0.02) for ox-SWCNTs, and from 0.46 to 0.53 (±0.05) for OH-SWCNTs (Figure 1C *vs.* Figure 4D; Figure 1C *vs.* Figure 4F). These data suggested that macrophages could normally degrade ox- and OH-SWCNTs. When macrophages were stimulated with PMA, the increase of I_D_/I_G_ ratios was even prominent after 24 h, from 0.33 to 0.81 (±0.04) for ox-SWCNTs (Figure 1C and Figure 4D) and from 0.46 to 0.73 (±0.07) for OH-SWCNTs (Figure 1C and Figure 4F). The results suggested that PMA-induced respiratory burst in macrophages could accelerate the biodegradation of ox- and OH-SWCNTs, inducing more defect sites on SWCNTs. Meanwhile, we found that NAC pretreatment of cells could significantly attenuate the biodegradation of ox-SWCNTs and OH-SWCNTs, with the I_D_/I_G_ ratios returned to control levels (Figure 4D,F). No above-mentioned effect was observed for p-SWCNTs. Taken together, our data support that macrophages can normally degrade ox-SWCNTs and OH-SWCNTs and the process can be speeded up significantly by PMA-induced respiratory burst in macrophages, probably through the generation of large amount of ROS, and thus anti-oxidant treatment with NAC can efficiently block the process.

### 2.3. Biodegradation of SWCNTs in Respiratory Burst Enzymatic System in Vitro

**Figure 5:**
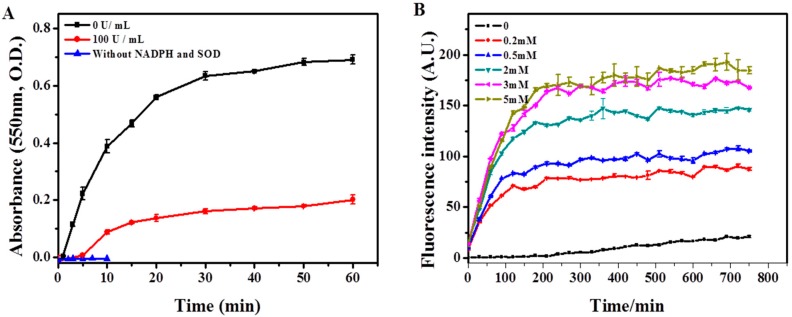
Construction of the *in vitro* respiratory burst enzymatic system, as evaluated through the generation of O_2_*^−^ and H_2_O_2_. (**A**) The generation of O_2_*^−^ by NOX solution over time in the presence or absence of SOD, as measured by ferrocytochrome c absorbance at 550 nm; (**B**) The production of H_2_O_2_ over time by NOX and SOD catalyses, in the presence of different concentrations (0–5 mM) of NADPH, as measured by the fluorimetric hydrogen peroxide assay.The data demonstrated that NOX was viable to produce O_2_*^−^, which was then converted to H_2_O_2_ by SOD.

To further confirm the role of respiratory burst in the degradation of SWCNTs, we constructed an enzymatic system to simulate the respiratory burst *in vitro* [54,55,56]. Respiratory burst is initiated by the activation of NOX (NADPH Oxidase), which is a complex enzyme consisting of five components including p40^PHOX^ (PHOX for Phagocyte oxidase), p47^PHOX^, p67^PHOX^, p22^PHOX^ and gp91^PHOX^. Once the cell is activated, the cytosolic subunits migrate to the membrane, where they bind to cytochrome b558 to assemble the active oxidase [57,58,59]. NOX catalyzes the generation of O_2_*^−^ from NADPH by transferring an electron to oxygen. Then the O_2_*^−^ is converted to H_2_O_2_ by superoxide dismutase (SOD). And the H_2_O_2_ serves as the substrate for peroxidase such as MPO in oxidating halides to generate hypohalous acids [44,60]. First, the construction of *in vitro* respiratory burst enzymatic reaction was evaluated by the measurement of O_2_*^−^ to test the activity of NOX. The NOX solution itself could not generate detectable amount of O_2_*^−^, manifested as constant low level of the absorption of ferrocytochrome C at 550 nm (Figure 5A). However, the absorption of ferrocytochrome C increased over time after the addition of NADPH into the NOX solution, and the increase could be significantly inhibited by SOD (100 U/mL), suggesting that NOX was viable to produce O_2_*^−^ after supplement of NADPH (Figure 5A). Next, we optimized the concentration of NADPH by measuring the production of H_2_O_2_ in the NOX + SOD system supplemented with different concentrations of NADPH, and results showed that addition of 5 mM NADPH generated the maximum amount of H_2_O_2_ (Figure 5B). The data indicated that the *in vitro* respiratory burst enzymatic system was constructed successfully and could generate required substrates (H_2_O_2_) for MPO.

**Figure 6:**
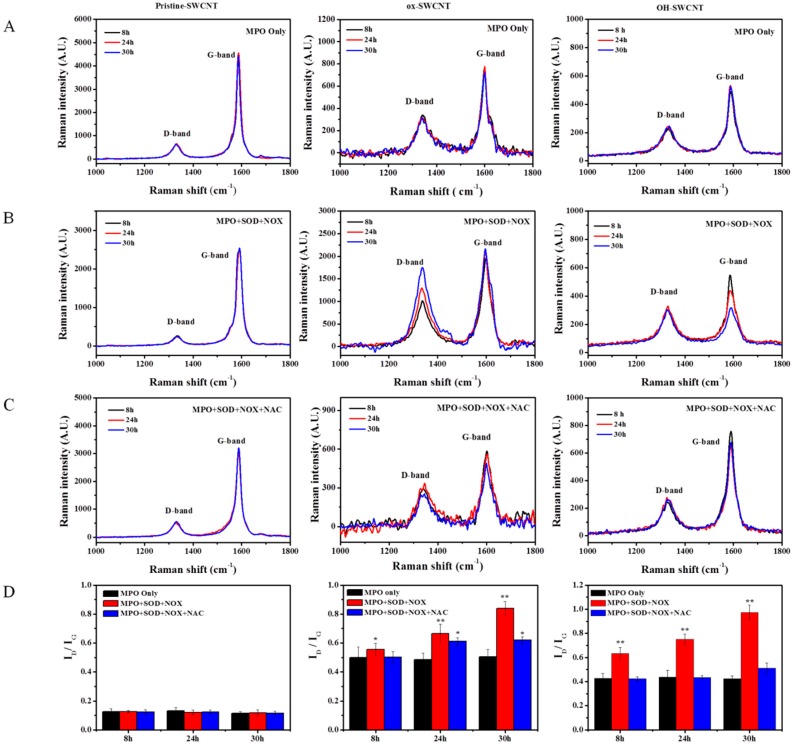
Degradation of SWCNTs by *in vitro* respiratory burst enzymatic system, as measured by Raman spectra. Column from left to right: pristine-SWCNTs, ox-SWCNTs and OH-SWCNTs (8 µg per sample). (**A**) Raman spectra of SWCNTs incubated with MPO alone (containing 140 mM NaCl, 1 U MPO, and 1 mM EGTA in PBS buffer) for the indicated time; (**B**) Raman spectra of SWCNTs incubated in full enzyme solution (labeled as MPO + SOD + NOX) for indicated time; (**C**) Raman spectra of SWCNTs in full enzyme solution supplemented with 1 mM NAC (labeled as MPO + SOD + NOX + NAC); (**D**) Corresponding quantitative measurement of I_D_/I_G_ ratios of SWCNTs incubated in (**A**–**C**). Results shown are the mean values ± S.D. of at least 20 spectra. Significance of differences by comparing with MPO only group was indicated with asterisks (* *p* < 0.05;** *p* < 0.01).

The nanotubes were previously shown to be degraded by hMPO in the presence of H_2_O_2_ [35,37,61]. hMPO can catalyze the production of even stronger oxidizing substances such as hypohalous acids in the presence of sodium chloride (NaCl) to degrade internalized particles and bacteria [44]. We therefore incubated SWCNTs in the *in vitro* enzymatic system with the addition of NaCl, and at different time points (8, 24, and 30 h) we sampled the SWCNTs to track the degradation process of SWCNTs. As shown in Figure 6, for p-SWCNTs, the Raman spectra have little or no change in the D-band and G-band intensity in MPO only, full enzyme solution (MPO + SOD + NOX) and NAC (MPO + SOD + NOX + NAC) groups, which means there is nearly no oxidation on p-SWCNTs. As for ox-SWCNTs and OH-SWCNTs, MPO alone caused no change in the Raman spectra, but in full enzyme solution, both types of SWCNTs exhibited the tendency of degradation as I_D_/I_G_ ratios increased with time (Figure 6, ox-SWCNTs, OH-SWCNTs), to 0.67 (±0.05) and 0.85 (±0.04) for ox-SWCNTs at 24 and 30 h, respectively, and to 0.76 (±0.04) and 0.93 (±0.06) for OH-SWCNTs at 24 and 30 h, respectively, suggesting that defect sites of SWCNTs increased and the structure of nanotubes were damaged. However, the degradation process could be suppressed by the addition of NAC to the full enzyme solution, as shown in Figure 6 (MPO + SOD + NOX + NAC group), proving that SWCNT biodegradation induced by respiratory burst could be completely inhibited (OH-SWCNTs) or alleviated (ox-SWCNTs) by anti-oxidants that scavenge the ROS.

**Figure 7:**
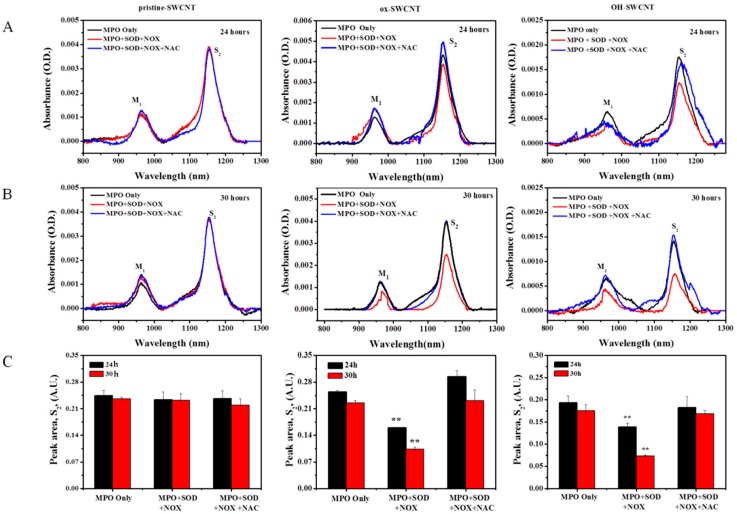
Degradation of SWCNTs by *in vitro* respiratory burst enzymatic system, as measured by UV-vis-NIR, normalized against baseline. Column from left to right: pristine-SWCNTs, ox-SWCNTs and OH-SWCNTs. The UV-vis-NIR spectra of SWCNTs incubated for 24 h (**A**) and 30 h (**B**) with different solutions were shown; (**C**) The peak area of S_2_ spectra of SWCNT. The reduced intensity of the S_2_ absorbance spectra indicating the degradation of SWCNTs. Results were from at least three samples and expressed as mean ± S.D. Significance of differences by comparing with the MPO only group was indicated with asterisks (** *p* < 0.01).

We further used UV-vis-NIR spectroscopy to assess the degradation of SWCNTs after 24 and 30 h incubation with enzyme solutions (Figure 7). Very similar to the results obtained from Raman spectrum analyses, we found essentially no change in the absorbance of S_2_ band of p-SWCNTs (Figure 7, pristine-SWCNTs). However, both ox-SWCNTs and OH-SWCNTs showed a significant decrease of the absorbance of S_2_ band in a time-dependent manner after being incubated with full enzyme solution, whereas the addition of NAC significantly inhibited the degradation process, with the peak area of S_2_ band back to the level comparable to that of MPO alone group (Figure 7C).

**Figure 8:**
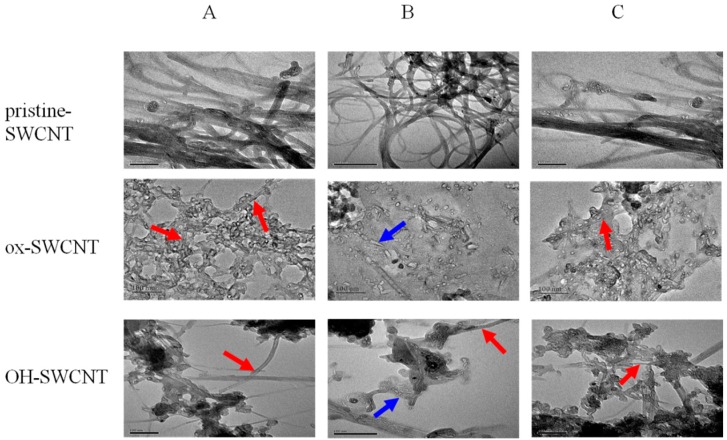
TEM imaging of SWCNTs (pristine-SWCNT, ox-SWCNT, OH-SWCNT) after 24 h incubation with *in vitro* respiratory burst enzymatic system. (**A**) MPO only; (**B**) Full enzyme solution (MPO + SOD + NOX); (**C**) Full enzyme solution supplemented with 1 mM NAC. Red arrows point to SWCNTs with long fiber-like shape; Blue arrows point to degraded fragments of SWCNTs. Scale bar = 100 nm.

The relatively simple composition of an *in vitro* enzymatic system allows us to examine the morphological change of SWCNTs as a result of enzymatic degradation by using transmission electron microscopy (TEM) (Figure 8). Before incubation in the enzymatic system, all original SWCNTs samples appeared to have a fiber-like shape, as shown in Figure 1. After 24 h incubation, no morphological change was observed for p-SWCNTs, which still exist as long-fiber shaped tubes (Figure 8, pristine-SWCNTs). For ox-SWCNTs and OH-SWCNTs, MPO treatment alone did not induce apparent change of SWCNTs, whereas in full enzyme solution, both SWCNTs underwent serious structural damage, resulting in fragmentation and loss of fibrous structure. Most of the tubular structure of SWCNTs became undefined. In addition, a small amount of debris and pieces of nanotubes were observed (Figure 8B). Taken together, our data obtained using an *in vitro* respiratory burst enzymatic system suggests that MPO alone could barely degrade SWCNTs; full respiratory burst enzymatic system could only degrade the ox-SWCNTs and OH-SWCNTs but not p-SWCNTs. More importantly, the degradation process can be modulated by NAC.

## 3. Discussion

The biodegradability of carbon nanotubes may be a major determinant of the toxic outcomes in exposed individuals [35]. Peroxidases such as MPO and EPO have been demonstrated to be able to degrade SWCNTs [35,36], due to their capability of converting H_2_O_2_ and oxidating halides to generate hypohalous acids [59,60,62]. In mammalian systems, macrophages are ubiquitous, patrolling for invading pathogens, dying cells and particles. Most often CNTs exposed to individuals were found engulfed by macrophages [41]. Thus, macrophages constitute the potential target cells for modulating CNTs biodegradation. The respiratory burst in macrophages activates a series of enzymatic reactions involving the enzyme NADPH oxidase, SOD (Cu/ZnSOD and MnSOD), and MPO that ultimately produce hypochlorous acid (HClO). Therefore, activation of respiratory bursts in macrophages by PMA would accelerate the biodegradation of internalized SWCNTs, as shown in this study. More prominently, our results also demonstrated that due to the intrinsic property of ROS production during the respiratory burst, an anti-oxidant treatment (such as NAC) of cells could efficiently suppress the degradation process.

Recently, the reactive intermediate produced by respiratory burst, O_2_*^−^, was also shown to react with nitric oxide (NO*) to generate a potent oxidant-peroxynitrite (ONOO^−^), which plays an important role in macrophage biodegradation of SWCNTs [63]. Therefore, PMA-induced respiratory burst in macrophages may also activate the peroxynitrite-driven biodegradation of SWCNTs. To further confirm the role of respiratory burst in the biodegradation of SWCNTs, we constructed an *in vitro* simulating enzymatic fluid of respiratory burst to rule out the interference of peroxynitrite. The simulating fluid could generate sufficient amounts of reactive intermediates including O_2_*^−^ and H_2_O_2_ (Figure 5), to provide substrates for MPO. Very similar to the results obtained from PMA-activated macrophages, the *in vitro* system containing full enzymes could considerably degrade ox-SWCNTs and OH-SWCNTs, manifested as increased I_D_/I_G_ ratios and decrease of S_2_ band. In addition, NAC supplemented to the system could cause apparent inhibition of the biodegradation of SWCNTs. These data suggested that respiratory burst played an important role in the biodegradation of SWCNTs, and the *in vitro* enzymatic system might be a useful tool to simulate the respiratory burst for other applications.

**Figure 9:**
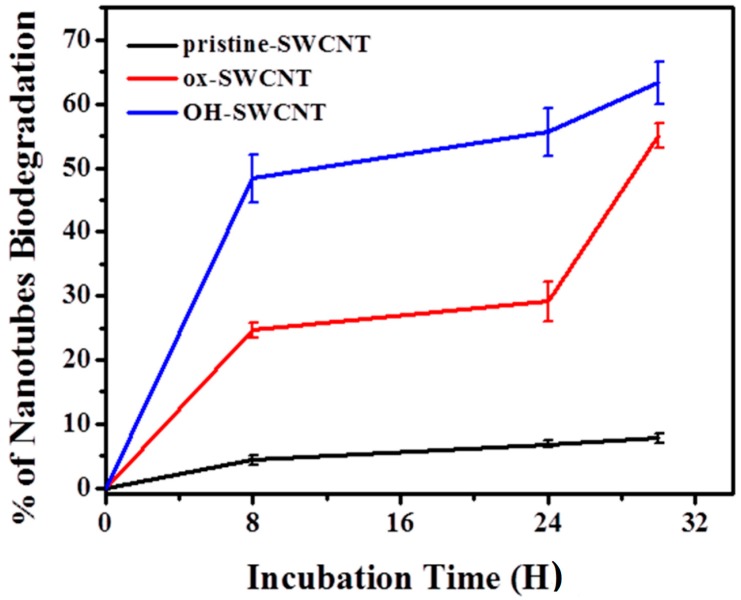
Biodegradation of SWCNTs by *in vitro* enzymatic system over time. The level of biodegradation at different time points is plotted as percentage assessed by the intensity magnitudes of the characteristic semi-conducting (S_2_) peaks of SWCNTs present in the recorded Vis-NIR spectra.

Previous studies showed a close relationship between the surface chemistry and the biodegradation of CNTs, and it seems that carboxylation on CNT surface is a prerequisite for CNT degradation [35]. The possible reason is because the carboxylation on CNTs might break the π-π interactions of CNTs and create two reactive sites that are potential sites for oxidative attack [64]. In this study, we compared three types of SWCNTs with different surface modifications, including p-SWCNTs, ox-SWCNTs, and OH-SWCNTs. The infrared spectra assessments revealed about 51% of OH-SWCNTs and 25% of ox-SWCNTs were degraded after 8 h incubation with an *in vitro* enzymatic system. After 30 h incubation, 63% of OH-SWCNTs and 53% of ox-SWCNTs were degraded. During the whole incubation, only negligible degradation (<5%) of pristine SWCNTs was observed (Figure 9). This suggests that except for p-SWCNTs carrying negligible defect sites, both ox-SWCNTs and OH-SWCNTs underwent serious structural damage and OH-SWCNTs were more susceptible to the oxidative attack than ox-SWCNTs. During the preparation of ox-SWCNTs, the acid oxidation of SWCNTs introduced both carboxyl and hydroxyl groups on the surface of SWCNTs, while OH-SWCNTs possess hydroxyl groups only (Figure 1B). It is difficult to differentiate the contribution of the carboxyl and hydroxyl groups to the biodegradation of SWCNTs. However, our results at least illustrated that oxygen-functionalized SWCNTs were biodegradable. Alternatively, the higher biodegradation of OH-SWCNTs may result from the fact that OH-SWCNTs originally possess higher level of defect sites than ox-SWCNTs do (Figure 1C), and thus provide more points for oxidative attack.

We observed 27% degradation of ox-SWCNTs after 24 h incubation with the *in vitro* enzymatic system (Figure 9). In another *in vitro* hMPO system reported by Kagan *et al.* [35], the degradation of IgG-coated oxidized SWCNTs seems to be much higher (solution turned translucent in 24 h), probably due to the constant higher concentrations of H_2_O_2_ kept throughout their experiments by frequent (every 5 h) replenishment of hMPO and H_2_O_2_. We envision that by increasing the frequency and concentration of NADPH and MPO added, higher degradation efficiency of the *in vitro* enzymatic system would be achieved. Interestingly, based on the absorbance of S_2_ band, the 24 h degradation of ox-SWCNTs in untreated macrophages is very similar to that reported by Kagan *et al.* [35] (25.8% in this study *vs.* ~20%). Compared with ox-SWCNTs, 35% of OH-SWCNTs were degraded under the same condition, further confirming that OH-SWCNTs were more biodegradable than ox-SWCNTs. With PMA treatment, macrophages degraded up to 67% of ox-SWCNTs in 24 h (Figure 3F), which is comparable to the efficiency of neutrophils that were shown to degrade ~30% of ox-SWCNTs (no IgG coating) within 12 h [35], suggesting that through proper stimulation, the biodegradation of SWCNTs by macrophages would be elevated to that by neutrophils.

Taken together, we have demonstrated both ways of accelerating and slowing down the biodegradation of SWCNTs in macrophages by modulating the respiratory burst. In addition, SWCNTs with defect sites were demonstrated to be more prone to be biodegraded by macrophages. Therefore, by careful functionalization of SWCNTs with proper coating that modulates the macrophage response, one can design better SWCNTs products with controlled retention time and toxicity. Alternatively, for drug delivery, modulating the biodegradation of nanocarriers may facilitate the release of the payloads as needed.

## 4. Materials and Methods

### 4.1. Materials

Pristine SWCNTs (p-SWCNTs, CNTs purity > 95%, SWCNT purity > 90%, ash < 5 weight %) and hydroxylated SWCNTs (OH-SWCNTs, purity > 90%) synthesized by chemical vapor deposition (CVD) method were originally obtained from Cheng du Organic Chemicals Co. (Cheng du, China). The following reagents were all purchased from Sigma (St. Louis, MO, USA): Phorbol myristate acetate (PMA), 2′,7′-dichlorofluorescein diacetate (DCF-DA), ferricytochrome c, β-nicotinamide adenine dinucleotide 2′-phosphate reduced tetrasodium salt hydrate(NADPH), bovine erythrocyte superoxide dismutase (SOD), EGTA, Flavin adenine dinucleotide disodium salt hydrate (FAD), Myeloperoxidase (MPO), *N*-acetyl-l-cysteine (NAC).

### 4.2. Preparation of SWCNTs Solutions and Characterization

Acid-oxidized SWCNTs (ox-SWCNTs) were prepared according to the procedure described previously [65,66,67]. In detail, 10 mg of SWCNTs were suspended in 40 mL of a 3:1 mixture of concentrated H_2_SO_4_/HNO_3_ in a 100 mL test tube and sonicated in a water bath (KQ-500DV, 100 kHz, 60%) for 24 h at 40–50 °C. The resultant suspension was then diluted with 200 mL deionized water and filtered through a membrane (pore size 0.22 µm), followed by wash with 50 mL deionized water on the membrane. The acid-oxidized nanotubes were re-suspended in deionized water at a concentration of 1 mg/mL with brief sonication (KQ-50DE, 40 kHz, 2 min). The resultant ox-SWCNT suspension was black, well dispersed and had a neutral pH. The stock solutions of 1 mg/mL pristine SWCNTs (p-SWCNTs) and hydroxylated SWCNTs (OH-SWCNTs) were prepared separately by mixing SWCNTs with cell culture medium, followed by sonication for 5 min on ice using a ultrasonic probe tip sonicator (JY92-IIN, Ningbo Scientz Biotechnology Co., Ltd. Ningbo, China).

For transmission electron microscopy (TEM) characterization, SWCNTs were diluted to 0.01 mg/mL and precipitated onto a copper net and dried for imaging with a Hitachi H-7500 transmission electron microscopy at 80 kv (Tokyo, Japan). In addition, the infrared spectra of SWCNTs were collected by using a FT-IR spectrometer (JASCO, Inc., Easton, MD, USA). Raman spectroscopy analyses were performed on an inVia Raman spectroscopy (Renishaw Plc., Gloucestershire, UK) with a 633 nm wavelength laser source.

### 4.3. Cell Culture and Treatment

The murine monocytic cell line Raw264.7 was obtained from American Type Culture Collection (ATCC: TIB-71) and cultured in a humidified incubator with 95% air and 5% CO_2_ at 37 °C in complete culture medium(cRPMI) containing RPMI-1640 fundamental medium and 100 U · mL^−1^ penicillin/streptomycin, supplement with 10% heat deactivated fetal bovine serum (FBS). All ingredients for the media were purchased from Hyclone Inc. (Waltham, MA, USA). For optimization of respiratory burst induction in macrophages, cells were seeded in a 96-well plate at a density of 2 × 10^5^ cells/mL and allowed to attach for overnight. Next, cells were loaded with 10 μM of 2′,7′-dichlorofluorescein diacetate (DCF-DA, a probe for intracellular ROS detection) at 37 °C for 30 min in dark. After wash with PBS twice, cells were treated with various concentrations (0–2 µM) of phorbol myristate acetate (PMA, a respiratory burst inducer [51,68]), and the fluorescence of DCF in cells was measured on a Thermo Varioskan Flash microplate reader (Winooski, VT, USA) at the excitation/emission wavelength 488 nm/530 nm very 10 s for the first 90 min and at time points of 3, 6, 9, 12, and 24 h. To optimize the concentration of *N*-acetyl-l-cysteine (NAC) for the inhibition of respiratory burst, cells were first pretreated with various concentrations (0–5 mM) of NAC for 1 h and then loaded with DCF-DA. After, cells were treated by PMA (0.5 µM) for 3 h in the presence of NAC (0–5 mM) and fluorescence detection was performed on the Varioskan Flash microplate reader. Furthermore, the DCF fluorescence in cells was also imaged with a Leica TCS SP5 confocal laser-scanning microscope (Mannheim, Germany) with a laser excitation at 488 nm and emission at 530 nm. For SWCNT treatment, cells (2 × 10^5^ cells/mL) were seeded in culture dishes (60 mm in diameter) and allowed to attach for overnight. Then, 15 µg of SWCNTs (p-SWCNTs, ox-SWCNTs, and OH-SWCNTs) were added to the cell culture along with PMA (0.5 µM). Cells with NAC pretreatment (1 mM), followed by the addition of PMA (0.5 µM) were set to investigate the inhibitory effect of antioxidant.

### 4.4. Construction of in Vitro Respiratory Burst Enzymatic System and Verification

First, NADPH oxidase (NOX) was isolated according to the method described previously [54,55,56]. In detail, the macrophages were suspended to a concentration of 0.5–1.5 × 10^8^ cells/mL in a phosphate-sucrose buffer (1× PBS buffer, pH 7.0, and 340 mM sucrose). Cell disruption was performed by 5 min sonication using an ultrasonic probe tip sonicator (JY92-IIN, Ningbo Scientz Biotechnology Co., Ltd., Ningbo, China) in a 1.5 mL tube on ice. Then, the homogenate was centrifuged at 800× *g* for 10 min at 4 °C to remove unbroken cells, nuclei, and large debris. The supernatant supplemented with EGTA (1 mM), and flavin adenine dinucleotide disodium salt hydrate (FAD, 0.1 mM) was used as the NADPH Oxidase (NOX). Thereafter, the second enzyme, SOD (300 U/mL) was added into the mixture, followed by the addition of NADPH (5 mM), NaCl (140 mM) and MPO (1 U). The full enzyme solution (total volume 500 µL) was then incubated at 37 °C with shaking (80 rpm) and replenishment of MPO (1 U) and NADPH (40 mM) every 8 h in dark. For SWCNTs incubation, 8 µg of SWCNTs per sample were mixed with the enzyme solution for indicated time, and then the samples were washed by centrifugation at 3400 rpm for 1 h twice and re-suspended in deionized water before Raman, UV-vis-NIR and TEM characterization.

The efficiency of this enzymatic system was evaluated by monitoring the generation of O_2_*^−^ and H_2_O_2_ following the presence of NOX and SOD in the system, respectively. O_2_*^−^ was measured by the method of ferricytochrome c reduction, as described by Bellavite *et al.* [69]. In detail, the 60 µL NOX solution were mixed with 0.94 mL phosphate-sucrose buffer containing ferricytochrome c (0.1 mM) and freshly prepared NADPH solution (0.2 mM). The absorption of ferrocytochrome C, the product of ferricytochrome C reduction, was measured at 550 nm on a UV-visible spectrophotometer (Agilent 8453, Santa Clara, CA, USA). The H_2_O_2_ was detected by using a Fluorimetric Hydrogen Peroxide Assay Kit (Sigma, St. Louis, MO, USA), according to manufacturer’s instruction.

### 4.5. Assessment of SWCNT Degradation

Raman spectroscopy. For cell samples, macrophages incubated with SWCNTs were fixed using 4% paraformaldehyde (4% PFA) onto a glass bottom cell culture dish (Nest Scientific, Rahway, NJ, USA). For samples from *in vitro* enzymatic system, 30 µL of solution were dropped on a monocrystalline silicon wafer and allowed to dry overnight. An inVia Raman microscope spectrometer (Renishaw Plc., Gloucestershire, UK) with a 633 nm laser source was used for all samples, spectrum was obtained over the range of 800 to 1800 cm^−1^ to visualize D and G band intensity changes throughout the degradation process. All Raman spectra were recorded for 10 s, 10% laser power, using a 50 L objective. For each sample, at least 20 spectra were recorded.

Infrared spectroscopy (UV-vis-NIR). For cell samples, macrophages incubated with SWCNTs were harvested together with the incubation medium. The cell suspensions were centrifuged (3400 rpm, 1 h) and resuspended in deionized water. Samples were further subjected to sonication for 20 min using a ultrasonic probe tip sonicator (JY92-IIN, Ningbo Scientz Biotechnology Co., Ltd. Zhejiang, China), and washed twice with PBS by centrifugation at 3400 rpm for 1 h to remove the cellular components before assessment of SWCNT degradation. The UV-vis-NIR spectra were acquired by using a Cary 5000 UV-Vis-NIR spectrophotometer (Varian, Palo Alto, USA).

Transmission Electron Microscopy (TEM). SWCNTs were mixed with the enzyme solution for 24 h, and the samples then were washed by centrifugation at 3400 rpm for 1 h twice and resuspended in deionised water. Five µL of prepared sample were precipitated onto a copper net and dried for imaging with a Hitachi H-7500 transmission electron microscopy at 80 kv (Tokyo, Japan).

### 4.6. Statistical Analysis

The data were expressed as the mean ± S.D., and the difference between groups was evaluated using Student’s *t*-test, with the significance level set at * *p* < 0.05 or ** *p* < 0.01.

## 5. Conclusions

In the present study, we explored and compared ways of modulating the biodegradation of three types of SWCNTs (p-, ox-, and OH-SWCNTs) in macrophages and in an *in vitro* enzymatic system simulating respiratory burst. The results demonstrated that: (1) SWCNTs carrying defect sites (ox-SWCNTs and OH-SWCNTs) could be degraded by macrophages, whereas p-SWCNTs were resistant to biodegradation probably due to the lack of reactive sites for oxidative attack; the order of degradation rate is OH-SWCNTs > ox-SWCNTs >> p-SWCNTs; (2) The biodegradation of SWCNTs could be significantly accelerated by PMA-activation in macrophages, or inhibited by an anti-oxidant, NAC; (3) Respiratory burst played an important role in accelerating the biodegradation of SWCNTs, as revealed by using the *in vitro* enzymatic system simulating respiratory burst. In addition, the *in vitro* enzymatic system constructed in the present study was proven to be able to simulate the enzymatic reaction cycles of respiratory burst for the effective degradation of SWCNTs. In principle, the enzymatic system can be expanded to study the degradation of other nanomaterials. Our findings on the biodegradation of functionalized SWCNTs would help to design safer SWCNT consumer products that are biodegradable and less toxic to living organisms, as well as drug carriers with controllable release of its payload. The ways of modulating the biodegradation of SWCNTs as we revealed might also provide invaluable clues on the development of intervention measurements for relieving the side effects of SWCNTs.

## Supplementary Materials

Supplementary materials can be found at http://www.mdpi.com/1422-0067/17/3/409/s1.

## Abbreviations

SWCNT: Single-walled carbon nanotube
HRP: Horse radish peroxidase
MPO: Myeloperoxidase
EPO: Eosinophil peroxidase
NETs: Neutrophil extracellular traps
PMN: Polymorphonuclear neutrophils
O_2_*^−^: Superoxide anion
NOX: Nicotinamide adenine dinucleotide phosphate (NADPH)-oxidase
SOD: Superoxide dismutase
PMA: Phorbol myristate acetate
LPS: Lipopolysaccharide
NAC: *N*-acetyl-l-cysteine
ROS: Reactive oxygen species
DCF-DA: 2′,7′-Dichlorofluorescein diacetate
TEM: Transmission electron microscopy
UV-vis-NIR: Ultraviolet-visible-near-infrared
FAD: Flavin adenine dinucleotide disodium salt hydrate
