# Supplementary material for: Biodegradation of Single-Walled Carbon Nanotubes in Macrophages through Respiratory Burst Modulation
Source: Int J Mol Sci. 2016 Mar 22;17(3):409. doi: 10.3390/ijms17030409 (PMC4813264; doi:10.3390/ijms17030409)
Supplement: Supplementary file 1 [file ijms-17-00409-s001.pdf]

Supplementary Materials: Biodegradation of Single-Walled Carbon Nanotubes in Macrophages through Respiratory Burst Modulation

Jie Hou, Bin Wan, Yu Yang, Xiao-Min Ren, Liang-Hong Guo and Jing-Fu Liu

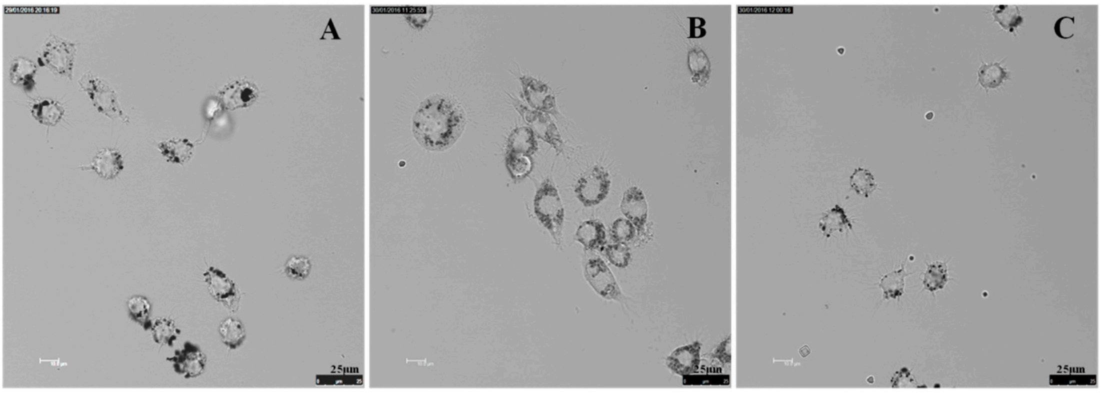

Figure S1. Bright-field images of the SWCNTs in macrophages demonstrate the internalization of CNTs within cells. (**A**) p-SWCNTs; (**B**) ox-SWCNTs; (**C**) OH-SWCNTs (5 μg/mL, 6 h). Scale bar = 25 μm.

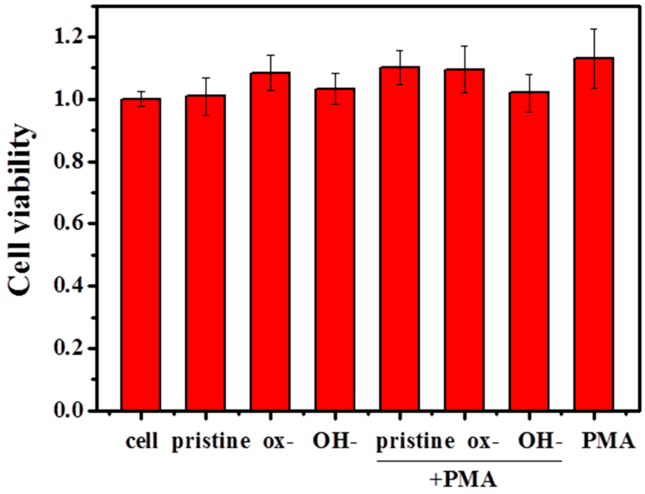

Figure S2. Viability of macrophages under 24 h exposures of different types of SWCNTs (pristine, ox- and OH-SWCNTs, 5 μg/mL) with or without PMA (0.5 μM) activation, as measured by WST-1 assay (Roche Applied Science, Bavaria, Germany). Cells were seeded on a 96-well plate and allowed to attach for overnight, after the treatment, 100 μL of WST-1 work solution were added into each well and incubated for 2 h at 37 °C, followed by reading absorbance at 490 nm (690 nm for background subtraction).

Table S1. Characteristics (length and diameters) of the SWCNTs before biodegradation.

	Pristine SWCNT [a]	ox-SWCNTs [b]	OH-SWCNTs [a]
Length	5–30 μm	630 ± 47 nm	5–30 μm
Diameter	1–2 nm	1–2 nm	1–2 nm

[a] Data from supplier (Chengdu Organic Chemicals Co., Ltd,. Chinese Academy of Sciences, Chengdu, China); [b] ox-SWCNTs were prepared by acid-oxidation of the pristine SWCNTs, and the diameter and length were measured by analyzing 100 individual CNTs in representative TEM images.
